# Self-Reported Health-Related Quality of Life and Residual Symptoms among Virologically Suppressed People Living with HIV in the Era of Single-Tablet Regimens in Taiwan: A Cross-Sectional Study

**DOI:** 10.3390/life14030294

**Published:** 2024-02-22

**Authors:** Chien-Yu Cheng, Hsiu-Yin Wang, Chia-Jui Yang

**Affiliations:** 1 Division of Infectious Diseases, Department of Internal Medicine, Taoyuan General Hospital, Ministry of Health and Welfare, Taoyuan 330, Taiwan; s841060@yahoo.com; 2 Institute of Public Health, School of Medicine, National Yang Ming Chiao Tung University, Taipei 112304, Taiwan; 3 Gilead Sciences, Taipei 110, Taiwan; hsiuyin.wang7@gilead.com; 4 Department of Internal Medicine, Far Eastern Memorial Hospital, New Taipei City 220, Taiwan; 5 School of Medicine, National Yang Ming Chiao Tung University, Taipei 112304, Taiwan

**Keywords:** health-related quality of life, symptom burden, virological suppression, single-tablet regimens, the fourth 90, people living with HIV

## Abstract

This study assessed the health-related quality of life (HRQoL) and residual symptom burden among virologically suppressed people living with human immunodeficiency virus (HIV) (PLWH) using a single-tablet regimen in Taiwan. This cross-sectional study administered a self-reported online survey between July and October 2021 to anonymised virologically suppressed PLWH aged ≥20 years. Demographic, HIV-related variables, EuroQol-5-dimensions (EQ-5D), visual analogue scale (VAS), and HIV Symptom Index were analysed. Bivariate analyses were performed to compare HRQoL differences between PLWH and non-PLWH. Among 120 PLWH, 80.9% had HIV diagnosis for <15 years, median antiretroviral therapy (ART) duration of 7.0 years (Q1–Q3:4.0–11.0), and 62.5% had ≥1 comorbidity. The most common comorbidities were depression (26.7%) and hyperlipidaemia (15.8%). About one-fifth of PLWH received constant family support (25.8%) and peer support (21.7%). Married individuals or individuals with higher incomes had significantly better family support status. There was no significant difference across the five dimensions between PLWH and non-PLWH. PLWH perceived being bothered by fatigue/lack of energy (63.3%), sleep difficulties (63.3%), feeling sad/low/unhappy (51.7%), and appearance changes (51.7%). PLWH could achieve similar HRQoL as non-PLWH with stable treatment, highlighting an opportunity to focus on person-centred holistic care beyond HIV, especially on the psychological aspect, for the best possible HRQoL for PLWH.

## 1. Introduction

There were approximately 28.7 million people living with human immunodeficiency virus (HIV)/acquired immunodeficiency syndrome (AIDS) receiving antiretroviral therapies (ARTs). The global ART coverage was approximately 75% in 2021 [1]. Antiretroviral therapies have prevention benefits, with evidence proving that virologically suppressed people living with HIV (PLWH) will not transmit HIV to their partners through sexual intercourse [2], further supporting the transition of HIV to a chronically manageable condition [3].

As the Global Health Sector Strategy targets of 90-90-90 expired in 2021, the Joint United Nations Programme on HIV/AIDS (UNAIDs) released updated targets in 2020 to support the health priority of preventing HIV-related mortality and morbidity in all population subgroups. The 95-95-95 targets sought to achieve 95% of PLWH to be diagnosed; 95% of diagnosed PLWH to have access to sustained ART treatment; and 95% of PLWH being treated to achieve viral suppression by 2025 [4]. Over the years, the global incidence of new HIV infections decreased [5,6], and the global target achieved at the end of 2020 was 84-87-90.

While the fast-track HIV strategy sought to end AIDS by 2030, it failed to consider the challenges associated with the needs and quality of life of PLWH as HIV is progressively being considered a chronic disease. According to the World Health Organization (WHO), quality of life is defined as an individual’s sense of well-being and function in relation to their culture, personal aspirations, and concerns [7]. The concept of how a disease or treatment would impact the physical, psychological, and social aspects of the quality of daily life is known as the health-related quality of life (HRQoL) [8].

Challenges to achieving a good quality of life among PLWH included physical (e.g., underlying comorbidities, non-communicable diseases, pain, and adverse drug reactions), psychological (e.g., depression, anxiety, and sleep disturbances), and socioeconomic aspects (e.g., education, societal discrimination, family/social support, and financial stress) [9,10,11,12,13]. These factors may lead to treatment discontinuation and virologic failure and ultimately impact the quality of life negatively. As HIV is becoming a manageable chronic condition, there is a pressing need to adopt a person-centric chronic care for PLWH, a “fourth 90”. This goes beyond the existing strategy of achieving sustained viral suppression to ensure that 90% of virologically suppressed PLWH have a good quality of life, encompassing comorbidities and self-perceived quality of life [9,14].

Patient-reported symptoms may also influence treatment adherence and increase the risk of viral load resurgence, disease progression, and lower quality of life among PLWH with sustained virologic suppression [15,16]. Symptoms such as pain, tiredness, depression, appetite loss, and sleep disorders have been found to be highly prevalent among PLWH [11,13,16,17] and could lead to patient distress. In the context of chronic conditions, inadequate assessment and management of symptoms can have adverse outcomes on patient care and lead to deleterious impacts on overall function and HRQoL [17,18]. Therefore, understanding the symptom burden would be key to quality patient-centric care in the HIV patient journey.

The introduction of single-tablet ART regimens (STRs) that are effective, easy to take, reduce side effects, and affordable has further facilitated the chronic management of HIV/AIDS [19,20]. STRs have been associated with higher patient adherence, translating to viral suppression, lower hospitalization rates, and better patient-reported outcomes as compared with multi-tablet regimens [20,21,22]. However, studies assessing the HRQoL of virologically suppressed PLWH were limited in the era of STRs, with most evaluating the impact of initiating STRs or switching to STRs [20,22,23,24], with one study showing a significantly lower symptom burden 24 weeks after switching to STRs [23].

According to the Taiwan Centers for Disease Control and Prevention (CDC), Taiwan exceeded the global numbers by reaching 90-93-95 in 2020 [25] and subsequently 90-94-95 in 2021 [26]. The HIV incidence in Taiwan has decreased from 10.7 per 100,000 persons in 2017 to 4.6 per 100,000 persons in 2022 [27,28], with fewer newly diagnosed HIV cases recorded in 2022 (1075 cases) than in 2021 (1247 cases) [29], suggesting a favourable outcome of Taiwan’s policies on HIV care. STRs were recommended as first-line treatment for treatment-naive PLWH and included in the Taiwanese reimbursement regulation since 2016 [24], but little is known about the HRQoL and if there remained any residual symptom burden of virologically suppressed PLWH on STRs. Therefore, this study aimed to assess the HRQoL and the associated factors that might impact HRQoL, such as residual HIV symptom burden, by patient self-reported questionnaires in virologically suppressed PLWH in the era of STR in Taiwan. The insights from this study would better support the optimization of HIV care policies for virologically suppressed PLWH in Taiwan.

## 2. Methods

### 2.1. Study Design

This is a cross-sectional, patient-reported outcome study conducted in Taiwan using an anonymous, self-administered online survey among virologically suppressed PLWH to explore the HRQoL, HIV symptom burden, and ART adherence. Cognitive interviews among PLWH will guide the development of the questionnaire to validate the instrument and assess the understanding of the survey materials by the respondents. The purpose of the cognitive interviews was to test the wording of the questionnaire and uncover underlying biases or desirability that may influence their responses. The wordings of the final questionnaire were revised appropriately based on the feedback from the cognitive interviews prior to administering it to the respondents.

General population data were extracted from the 2020 Taiwan National Health and Wellness Survey (NHWS), an Oracle Life Sciences (formerly Cerner Enviza) internal patient-reported outcome database and compared with the HRQoL from the study. The NHWS is a cross-sectional survey conducted across 11 countries, including the United States, Europe (France, Germany, Italy, Spain, and the United Kingdom), Brazil, China, Japan, South Korea, and Taiwan. The survey collects self-reported characteristics, disease status, treatment use, and patient-reported outcomes (including HRQoL instruments such as the EuroQol health state questionnaire) from adult respondents aged 18 years or older in the countries where the survey was administered. Only deidentified responses from those who had provided informed consent were collected and analysed.

The PLWH-specific study protocol and questionnaire (#21-KANT-258) and the 2020 NHWS survey (#19-KANT-211) were reviewed and were granted exemption status and approved by the Pearl Pathways Institutional Review Board.

### 2.2. Study Population

People living with HIV aged 20 years or older, who have received ART for at least 2 years, and whose latest viral load was less than 200 copies/mL within one year before the survey. Participants who had provided informed consent and agreed to complete the survey and had not participated in any quality-of-life survey in a month prior to the study were recruited to the study. The participants were recruited from six patient advocacy groups (PAGs) in Taiwan between 19 July and 15 October 2021. Only deidentified responses were collected and analysed in this study.

An anonymised dataset of non-PLWH respondents was extracted from the existing 2020 Taiwan survey. Non-PLWH respondents were identified from a cohort of individuals from adult respondents of the general population who participated in the 2020 Taiwan NHWS. The adult NHWS respondents were recruited through an existing, general-purpose web-based consumer panel and had explicitly agreed to participate in the Taiwan NHWS in 2020. Recruitment to the 2020 Taiwan NHWS was based on random sampling and stratified by age and sex to ensure that the demographic composition is representative of the adult population in Taiwan. The dataset of NHWS respondents who had a self-reported diagnosis of HIV was excluded from this study.

### 2.3. Variables and Outcomes

#### 2.3.1. Demographic and HIV-Related Variables

Demographic data included age, gender, residential area, education level, employment status, annual income, marital status, and comorbidity.

HIV-related information, such as year of HIV diagnosis, duration of ART, frequency of hospital visits, viral load testing viral load results, and support status from family, peers, or society, were collected [30].

#### 2.3.2. Health-Related Quality of Life

The HRQoL measures were determined by the 5-level EuroQol-5-dimensions (EQ-5D-5L) questionnaire. The EQ-5D-5L instrument is a standardised measure of health status that comprises five dimensions: mobility, usual activities, self-care, pain/discomfort, and anxiety/depression [31]. Each dimension has five levels: no problems, slight problems, moderate problems, severe problems, and extreme problems. A 0–100 visual analogue scale (VAS) was also used to assess the health status on the day of the survey, wherein “0” corresponded to “the worst health state you can imagine” and “100” corresponded to “the best health state you can imagine”.

#### 2.3.3. HIV Symptom Index (HIV-SI) Questionnaire

The self-reported HIV-SI questionnaire comprised 20 items of HIV symptoms based on their occurrence within the past four weeks [32]. Each symptom has five levels: no symptom, have symptom but not bothersome, a little bothersome, very bothersome, and extremely bothersome. The level of bother from HIV symptoms was dichotomised into “not bothersome” (0—no symptom; 1—not bothersome) and “bothersome” (2—a little bothersome, 3—very bothersome, and 4—extremely bothersome) [30].

#### 2.3.4. Treatment Adherence

A 0-100 VAS was used to evaluate the amount of ART received in the past 30 days, whereby 0% indicated that the PLWH respondent did not take any ART, while 100% indicated that the PLWH respondent had taken all of the ART prescribed. The questionnaire also measured the number of days that PLWH missed taking their ART medication in the past 30 days and seven days, respectively.

### 2.4. Statistical Analyses

Continuous data was described with counts (numbers), mean (±standard deviation [SD]), and median (interquartile range [IQR], minimum, maximum, and percentiles), while categorical data were described by frequencies and percentages.

Bivariate logistic regression models were used to evaluate the association between the baseline characteristics of PLWH (independent variable) and support status (dependent variable). The support status was dichotomised into two outcomes in the logistic regression—“non-supportive” (0—never support; 1—seldom support; 2—somewhat support) and “supportive” (3—usually support; 4—always support). Variables with statistically significant association on bivariate logistic regression (*p* < 0.05) were included in a multiple logistic regression model to identify statistically significant covariates from all potential covariates.

To investigate how the HRQoL (EQ-5D and EQ-VAS) may differ between PLWH and non-PLWH, any differences in HRQoL between PLWH and non-PLWH were analysed using 1:1 propensity score (PS) matching based on sociodemographic characteristics via a greedy matching algorithm. The PS matching was used to address any potential confounders in respondent characteristics and unequal sample size between PLWH and non-PLWH. Sociodemographic-related covariates assessed in the matching included age, gender, residential area, education level, marital status, and income level. Bivariate comparisons of health-related outcomes between PLWH and PS-matched non-PLWH were performed using chi-squared tests.

Statistical significance was assessed at a significance level of *p*-value < 0.05. All data analyses were conducted using IBM SPSS Statistics for Windows, version 25 (IBM Corp., Armonk, NY, USA).

## 3. Results

### 3.1. Patient Demographic and Clinical Characteristics

A total of 120 virologically suppressed PLWH was recruited. The median age of PLWH was 30.3 (IQR: 22.0–38.0) years. Most PLWH were males (n = 113, 94.2%), living in urban areas (n = 98, 81.7%), single (n = 104, 86.7%), and had an annual income of less than USD 33,809 (n = 105, 87.5%). About half of PLWH lived in Northern Taiwan (n = 60, 50.0%), had a university degree (n = 85, 70.8%), and were employed with a full-time job (n = 62, 51.7%) (Table 1).

Among PLWH, 80.9% (n = 97) were diagnosed with HIV for less than 15 years, with a median duration of ART of 7.0 (IQR: 4.0–11.0) years (Table 1). The majority of the PLWH visited the HIV clinic every three months in the past year (n = 96, 80.0%), had their most recent HIV viral load test within 6 months (n = 104, 86.7%), and had undetectable viral load (n = 104, 86.7%). Among PLWH with at least one comorbidity (n = 75, 62.5%), the most common mental and physical comorbidities were depression (n = 32, 26.7%) and hyperlipidaemia (n = 19, 15.8%), respectively (Table 1).

### 3.2. Symptom Burden

The mean (SD) number of HIV symptoms reported by PLWH is 10.5 (5.4), and the mean (SD) number of bothersome symptoms is 6.5 (4.6) (Table 2). A cut-off age of 40 years old was used to explore the symptom burden between PLWH aged <40 years (n = 102) and PLWH aged ≥40 years (n = 18). PLWH aged ≥40 years had a numerically higher number of bothersome symptoms (mean: 7.4; SD: 6.2) than PLWH aged <40 years (mean: 6.4; SD: 4.3) (Table 2). Out of 20 symptoms in the HIV-SI, four symptoms were reported as bothersome by more than half of PLWH: fatigue or lack of energy (63.3%), difficulty falling asleep or remaining asleep (63.3%), feeling sad, low, or unhappy (51.7%), and changes in appearance (51.7%) (Supplementary Table S1).

### 3.3. Adherence

In the past month of the survey conducted, the median self-reported adherence was 100.0 (IQR: 99.0–100.0). Nearly half of the PLWH respondents (45.0%, n = 54) had missed at least one day of ART in the past month, and 20.8% (n = 25) had missed at least one day of ART in the past week (Table 3).

### 3.4. Support Status

The proportion of PLWH respondents who received constant support from family was 25.8% (n = 31), from their peers was 21.7% (n = 26), and from society was 11.7% (n = 14) (Table 4).

Among the PLWH respondents, individuals who were married (adjusted odds ratio [aOR] = 12.8; *p* = 0.02) and had higher incomes (aOR = 2.7; *p* = 0.03) had significantly better family support status. There were no significant differences between the patient characteristics and peer support status or social support status after adjusting all factors (Supplementary Tables S2–S4).

### 3.5. Health-Related Quality of Life among PLWH and PLWH vs. Non-PLWH

The median EQ-VAS score of PLWH was 80.0 (IQR: 69.3–90.0). The majority of PLWH self-reported having no problem on health profile in terms of mobility (n = 108, 90.0%), self-care (n = 119, 99.2%), and usual activities (n = 107, 89.2%). More than one-third of PLWH felt slight to severe pain/discomfort (n = 44, 36.7%) and slight to extreme problems on the anxiety/depression dimension (n = 58, 48.3%) (Table 5).

One PLWH respondent was excluded from 1:1 PS matching due to a missing response to the sex-identifier question. After 1:1 PS matching, a total of 119 PS-matched non-PLWH respondents were identified from 19,964 NWHS respondents (self-reported without HIV) (Supplementary Table S5).

The health quality across five dimensions of the EQ-5D-5L was not statistically different between PLWH and PS-matched non-PLWH respondents. A numerically higher proportion of PLWH (48.0%) than non-PLWH (35.0%) reported having slight to extreme problems in the anxiety/depression dimension. In terms of EQ-VAS, PLWH had a significantly lower VAS mean (±SD) score than PS-matched non-PLWH respondents (76.9 ± 17.8 vs. 81.3 ± 16.1; *p* = 0.048) (Table 6).

## 4. Discussion

With the advances in HIV management, the life expectancy of PLWH in recent decades has reached a similar level to that of the general population [34,35]. This study is the first in Taiwan to assess the HRQoL of virologically suppressed PLWH who were on STRs. The findings found that the health state quality of life among PLWH on STR with sustained virological suppression was similar to PS-matched non-PLWH. Simultaneously, our study identified that there remains an unmet need for more holistic HIV care to support PLWH in Taiwan even when they achieved virological suppression.

The observation of similar HRQoL between PLWH and non-PLWH in this study contrasted with previous local [13,36,37] and global studies [38,39,40], wherein HIV infection was associated with a more substantial negative impact on the HRQoL. As the treatment advancement evolves, PLWH respondents in this study were on STR and had sustained viral load suppression; the findings support the notion of a favourable outcome of Taiwan’s current HIV care system.

Of note, virologically suppressed PLWH, globally, still suffered from physical and mental comorbidities associated with HIV [9,41]. Similarly seen in this study, residual disease burden was observed in PLWH despite being on stable treatment and virologically suppressed. More than one-third of the PLWH respondents reported less satisfaction with pain/discomfort and anxiety/depression on EQ-5D. This was consistent in most studies where pain had been found to be a debilitating issue among HIV patients [39,42,43] and associated with increased impairments in physical function [43,44]. Anxiety and depression were also frequently reported by PLWH, contributed by stigma and discrimination from family, friends, or even healthcare providers [13,40,45,46]. Being fatigued or lack of energy, sleep-related issues, feeling sad, low, or unhappy, and changes in appearances were most mentioned as bothersome among virologically suppressed PLWH in this study, consistent with the HIV symptom burden reported globally [11,13,16,17].

Studies have shown that concerns about appearance-related side effects underlay psychological distress, depression, and ART non-adherence among PLWH [47,48]. Sleep-related issues, such as insomnia or hypersomnia, usually associated with extreme fatigue, were also common among virologically suppressed PLWH or long-term ART [49,50,51]. HIV-related sleep disturbances have been linked to psychological disturbances and pain [52,53]. Considering that some HIV-related symptoms (appearance change and sleep disturbances) could be attributed to ART [48,49,50,54], it may be of interest to further understand if potential side effects of ARTs could contribute to the symptom burden of HIV and intervene as appropriate [55]. Collectively, these suggest a need to assess factors contributing to and reducing residual symptom burden in PLWH with sustained viral suppression. It is, therefore, crucial to integrate care to address the residual HIV-related symptoms to prevent negative impact on the overall quality of life and effective management of HIV.

The self-reported ART adherence in this study was found to be high, possibly owing to Taiwan’s policy for HIV, wherein individuals diagnosed with HIV are provided free access to ART under the national health insurance and the case management system, which provides support to PLWH in Taiwan [56,57,58]. Reducing the number of ART pills to a single-tablet regimen was found to improve adherence and quality of life among PLWH [19,21]. This could also potentially explain the comparable HRQoL between PLWH and non-PLWH. However, there was still a substantial proportion of respondents who had self-reported missing doses, suggesting a possible gap in HIV care. This finding suggests that continuous adherence counselling/reminding may still be warranted from a chronic disease management perspective [58].

Support systems and strategies are essential for PLWH to facilitate positive coping mechanisms to manage challenges associated with HIV, including but not limited to residual disease burden, and boost treatment adherence [59,60]. Significant positive associations were observed between being married/having support and medication adherence [10,13,61], signifying the role of companionship for HIV care and support for a better quality of life. A higher level of family support was associated with being married or living with a partner and higher income in this study. Studies have shown that PLWH with higher incomes were more likely to have family support compared to those with lower incomes [62,63]. This could be attributed to reduced economic insecurity and objective support among households with higher income [63,64], spotlighting a need for further attention to improve support systems among single and/or low-income PLWH.

Notably, social support had been rated as less supportive by PLWH in this study than family or peer support, and about one-fifth of PLWH reportedly did not receive any support. As PLWH live longer, there is an increasing complexity associated with not only managing comorbidities but also the socioeconomic status, healthcare access, health literacy, and perceived HIV stigma and discrimination among PLWH [41]. The impact of HIV-related stigma could explain the reduced social support observed in this study [59,65] despite achieving virological suppression. In this regard, it is imperative to provide support from the community and healthcare providers to PLWH, improve inclusivity, and build stigma-reduction programs to dispel stigma between PLWH and the community in Taiwan to allow better coping with HIV [66,67]. Tactics to promote mental health as well as recovery and rehabilitation programs should be designed in consideration of specific cultural contexts, i.e., in the aspects of medical practice, geography, ethnicity, and religion [67]. For instance, multiple initiatives using the collaborative care model established in South Africa outlined the roles of the community and capacity to build an integrated care delivery for PLWH through primary care mental health, psychotherapy, and knowledge empowerment [67]. In Thailand, a 12-month culturally relevant behavioural intervention among PLWH successfully improved the quality of life, wherein depression and internalised stigma were reduced after the intervention [68].

Overall, this study provided insights into the overall HRQoL among PLWH in Taiwan who were on stable treatment and had achieved virological suppression in the era of STRs. The respondents living with HIV were found to have similar HRQoL as the general population in this study, which insinuates effective HIV care management in Taiwan. However, there are still unmet needs, in physical and psychological aspects, evident from the residual symptom burden among PLWH who had achieved viral suppression while on STRs, which could negatively impact the HRQoL. This indicates a need to further improve HIV care and management, address the HIV symptom burden, and strengthen social support among PLWH to achieve the “fourth 90” in Taiwan.

There are limitations in this study. The online outreach of the questionnaire may be limited by its required access to the internet or internet-based technologies, and thus, the study result may not be representative of those without technological literacy (e.g., the elderly or institutionalised individuals) in Taiwan. Additionally, social desirability bias may remain despite using cognitive interviews to assess the wording of the questionnaire, as respondents may feel uncomfortable responding to sensitive-related questions and be self-pressured to respond positively. This risk may be minimised by the use of self-reported questionnaires, which could boost responsiveness and involvement due to the greater anonymity and confidentiality that could overcome concerns of stigma and discrimination associated with HIV. Furthermore, the greater anonymity and privacy of online-based surveys than face-to-face data collection methods may increase the willingness and comfort of PLWH as they may not be willing to present or disclose their HIV status to the interviewers during the survey.

While the study findings provided valuable insights into the HRQoL and symptom burden of virologically suppressed PLWH in Taiwan, the sample population of 120 PLWH respondents may not represent the overall PLWH population across Taiwan. First, it could be noted the age distribution of PLWH in this study was similar to HIV-diagnosed Taiwanese patients between 2010 and 2013, where the mean age was 36.6 years and 34.1% were aged 40 years and older [69]. However, compared to the earlier study, a higher proportion of the respondents (n = 102 of 120, 85.0%) in this study were younger than 40 years old. Second, the respondents were recruited through their affiliations with PAGs, with the majority living in urban parts of Taiwan, and respondents not affiliated with PAGs or living outside of urban Taiwan were not assessed in this study. Third, the male–female respondent ratio of the PLWH population in this study was higher than the sex-specific prevalence of PLWH in Taiwan (19:1 vs. 12:1) [56]. This could be due to constraints in reaching out to female PLWH respondents due to the sensitive nature of HIV infection [70], thus generalising the impact of HIV in a gender-specific manner. Overall, the findings could not be generalizable to all PLWH in Taiwan. Further research with a larger sample size and recruitment outside of PAGs would be warranted. Future studies among female PLWH are recommended to gain a more comprehensive understanding of the female-specific patient-reported outcomes and HRQoL for the holistic management of HIV.

While a 1:1 PS matching was adopted in this study to address any confounding variables between PLWH and non-PLWH, this was limited to the respondents’ baseline characteristics as consideration of external factors, e.g., the year of recruitment (PLWH vs. non-PLWH) was excluded. This limited the generalisation of HRQoL comparison as the data collection period for PLWH was in 2021 and non-PLWH was in 2020. Further studies involving a similar recruitment period would be warranted. Additionally, further analyses to elucidate factors associated with HRQoL (e.g., duration of HIV diagnosis or being on STRs) were not conducted in this study and would be valuable to investigate in future studies with a larger sample size of PLWH on STRs.

## 5. Conclusions

With the stable treatment and sustained virological suppression achieved in the era of STRs, PLWH can achieve an HRQoL similar to that of the general population. However, there remains a residual symptom burden and gaps in social support among virologically suppressed PLWH, which could negatively impact HRQoL. This highlighted the opportunity to focus more on person-centred medical care beyond HIV to achieve the best possible HRQoL and well-being for PLWH. Specifically, consideration of physical and psychological aspects of HIV care, as well as interventions to reduce symptom burden and strengthen social support, would need to be incorporated as a part of a holistic management program in future treatment of HIV/AIDS in Taiwan.

## Figures and Tables

**Table 1:** Demographic and clinical characteristics of PLWH respondents (n = 120).

		PLWH (n = 120)	
Age, Years	*Median (IQR)*	30.3 (22.0–38.0)	
		n	%
**Gender ^†^**	*Male*	113	94.2
	*Female*	6	5.0
**Residential area**	*Northern*	60	50.0
	*Central*	31	25.8
	*Southern*	15	12.5
	*Eastern and others*	14	11.7
**Residential type**	*Urban*	98	81.7
	*Rural*	22	18.3
**Highest education level**	*Junior high school or less*	13	10.8
	*Senior high school/vocational school or an equivalent education level*	22	18.3
	*University or above*	85	70.8
**Employment status**	*Full-time employment*	62	51.7
	*Part-time employment*	17	14.2
	*Self-employed*	5	4.2
	*Unemployed (including home keeping/retired/student)*	27	22.5
	*Decline to answer*	9	7.5
**Marital status**	*Married/living with partner*	12	10.0
	*Single (never married/divorced/widowed)*	104	86.7
	*Decline to answer*	4	3.3
**Annual income ^‡^**	*Less than USD 3381*	43	35.8
	*USD 3381~USD 16,904*	38	31.7
	*USD 16,904~USD 33,809*	24	20.0
	*USD 33,809~USD 50,713*	5	4.2
	*Decline to answer*	10	8.3
**Disease duration**	*2–5 years*	35	29.2
	*5–15 years*	62	51.7
	*15–25 years*	22	18.3
	*More than 25 years*	1	0.8
**ART duration, years**	*Median (IQR)*	7.0 (4.0–11.0)	
**Hospital visits due to HIV (P1Y)**	*Once per month*	13	10.8
	*Every two months*	3	2.5
	*Every three months*	96	80.0
	*Every six months*	7	5.8
	*More than six months*	1	0.8
**HIV viral load testing**	*Within 6 months*	104	86.7
	*Between 6 and 12 months*	16	13.3
**HIV viral load result**	*With viral load, but less than 200 cells/mm* ^3^	16	13.3
	*Undetectable*	104	86.7
**Number of Comorbidities**	*0*	45	37.5
	*1*	31	25.8
	*2*	23	19.2
	≥*3*	21	17.5
**Comorbidity—Mental disease**	*Depression*	32	26.7
	*Generalised anxiety disorder*	15	12.5
	*Bipolar disorder*	11	9.2
**Comorbidity—Physical disease**	*Hyperlipidaemia*	19	15.8
	*Hypertension*	16	13.3
	*Hepatitis C*	14	11.7
	*Cardiovascular disease*	11	9.2
	*Diabetes mellitus*	9	7.5
	*Hepatitis B*	9	7.5
	*Osteoporosis*	6	5.0
	*Chronic liver disease*	5	4.2
	*Cancer*	2	1.7
	*Pneumonia*	1	0.8
	*Chronic kidney disease*	0	0.0

^†^ n = 1 respondent preferred not to answer; P1Y: past one year. ^‡^ Exchange rate USD 1 = 29.5780 NTD [33]).

**Table 2:** PLWH’s self-reported symptom burden (total n = 120).

HIV Symptoms per Patient	Mean	SD
**All patients (n = 120)**		
*Number of symptoms*	10.5	5.4
*Number of bothersome symptoms*	6.5	4.6
**Patient aged ≤40 y/o (n = 102)**		
*Number of symptoms*	10.5	5.5
*Number of bothersome symptoms*	6.4	4.3
**Patient aged >40 y/o (n = 18)**		
*Number of symptoms*	10.8	5.1
*Number of bothersome symptoms*	7.4	6.2

**Table 3:** PLWH’s self-reported adherence level (total n = 120).

Self-Reported ART Adherence Level				
**ART Adherence VAS score**	**PLWH**			
*Median (IQR)*	100 (99–100)			
**Forgotten days of medication**	**Past 1 month**		**Past 1 week**	
	**n**	**%**	**n**	**%**
*0 day*	66	55	95	79.2
*1 day*	28	23.3	18	15
*2 days*	10	8.3	5	4.2
*3 days*	9	7.5	0	0
*4 days*	3	2.5	0	0
*5 days*	1	0.8	1	0.8
*6 days*	1	0.8	1	0.8
*7 days*	0	0	-	-
*8 days*	2	1.7	-	-

**Table 4:** PLWH’s family/peer/social support (total n = 120).

Support Status	Family Support		Peer Support		Social Support	
	n	%	n	%	n	%
*Never support/not available*	23	19.2	9	7.5	14	11.7
*Seldom support*	15	12.5	14	11.7	27	22.5
*Somewhat support*	20	16.7	28	23.3	35	29.2
*Usually support*	31	25.8	43	35.8	30	25
*Always support*	31	25.8	26	21.7	14	11.7

**Table 5:** PLWH’s self-reported quality of life (EQ-5D-5L) (total n = 120).

EQ-5D-5L	PLWH (n = 120)									
**VAS Score**										
*Median (IQR)*	80.0 (69.3–90.0)									
**Dimensions of EQ-5D**	**No problem**		**Slight problem**		**Moderate problem**		**Severe problem**		**Extreme problem**	
	**n**	**%**	**n**	**%**	**n**	**%**	**n**	**%**	**n**	**%**
Mobility	108	90.0	9	7.5	2	1.7	1	0.8	-	-
Self-Care	119	99.2	1	0.8	-	-	-	-	-	-
Usual Activities	107	89.2	11	9.2	2	1.7	-	-	-	-
Pain/Discomfort	76	63.3	32	26.7	8	6.7	4	3.3	-	-
Anxiety/Depression	62	51.7	42	35.0	11	9.2	3	2.5	2	1.7

**Table 6:** Self-reported quality of life between PLWH (n = 119) and matched non-PLWH (n = 119).

EQ-5D-5L	PLWH (n = 119)	Matched Non-PLWH (n = 119)	*p*-Value
**VAS Score, mean (SD)**	76.9 (17.8)	81.3 (16.1)	0.048
**Dimensions of EQ-5D-5L**	%	%	
**Mobility**			0.905
*No problem*	90.0%	87.0%	
*Slight problem*	8.0%	10.0%	
*Moderate problem*	2.0%	2.0%	
*Severe problem*	1.0%	1.0%	
*Extreme problem*	0.0%	1.0%	
**Self-Care**			0.182
*No problem*	99.0%	95.0%	
*Slight problem*	1.0%	3.0%	
*Moderate problem*	0.0%	2.0%	
*Severe problem*	0.0%	1.0%	
*Extreme problem*	0.0%	0.0%	
**Usual Activities**			0.398
*No problem*	89.0%	92.0%	
*Slight problem*	9.0%	5.0%	
*Moderate problem*	2.0%	2.0%	
*Severe problem*	0.0%	1.0%	
*Extreme problem*	0.0%	0.0%	
**Pain/Discomfort**			0.125
*No problem*	64.0%	59.0%	
*Slight problem*	27.0%	37.0%	
*Moderate problem*	7.0%	2.0%	
*Severe problem*	3.0%	3.0%	
*Extreme problem*	0.0%	0.0%	
**Anxiety/Depression**			0.096
*No problem*	52.0%	65.0%	
*Slight problem*	35.0%	29.0%	
*Moderate problem*	8.0%	7.0%	
*Severe problem*	3.0%	0.0%	
*Extreme problem*	2.0%	0.0%	

## Data Availability

The original contributions presented in the study are included in the article/supplementary material, further inquiries can be directed to the corresponding author/s.

## Supplementary Materials

The following supporting information can be downloaded at: https://www.mdpi.com/article/10.3390/life14030294/s1, Table S1: HIV symptom burden perceived by PLWH respondents dichotomized into “bothersome” and “not bothersome; Table S2: Regression model of PLWH’s characteristics and Family support (Total n = 119); Table S3: Regression model of PLWH’s characteristics and Peer support (Total n = 119); Table S4: Regression model of PLWH’s characteristics and Social support (Total n = 119); Table S5: Demographics of PLWH and (pre-matched and post-matched) non-PLWH respondents.

## Abbreviations

AIDS	acquired immunodeficiency syndrome
aOR	adjusted odds ratio
ART	antiretroviral therapy
CDC	Centers for Disease Control and Prevention
EQ-5D-5-L	5-level EuroQol-5-dimensions
HIV	human immunodeficiency virus
HIV-SI	HIV Symptom Index
HRQoL	health-related quality of life
IQR	interquartile range
NHWS	National Health and Wellness Survey
PAG	patient advocacy groups
PLWH	people living with HIV
PS	propensity score
SD	standard deviation
STR	single-tablet ART regimen
UNAIDS	Joint United Nations Programme on HIV/AIDS (UNAIDs)
VAS	visual analogue scale
WHO	World Health Organization.
