# Supplementary material for: Self-Reported Health-Related Quality of Life and Residual Symptoms among Virologically Suppressed People Living with HIV in the Era of Single-Tablet Regimens in Taiwan: A Cross-Sectional Study
Source: Life (Basel). 2024 Feb 22;14(3):294. doi: 10.3390/life14030294 (PMC10971146; doi:10.3390/life14030294)
Supplement: Supplementary file 1 [file life-14-00294-s001.zip › life-2712877-supplementary.pdf]

Supplementary Table 1. HIV symptom burden perceived by PLWH respondents dichotomized into "bothersome" and "not bothersome".

HIV Symptoms burden	Bothersome[a]	Not bothersome[b]
	%	%
Fatigue or lack of energy	63.3	36.6
Difficulty of falling asleep or remaining asleep	63.3	36.7
Feeling sad, low or unhappy	51.7	48.3
Changes in appearance, such as accumulation of fat or weight gain	51.7	48.3
Feeling nervous or anxious	47.5	52.5
Skin problems, such as rashes, dryness, and itchiness	40.8	59.1
Memory difficulty	39.2	60.9
Sex problems, such as loss of sexual drive and dissatisfaction with sex life	38.3	61.7
Muscle or joint pain	29.2	70.8
Flatulence, stomach pain or gas in the stomach	28.3	71.7
Feeling pain, numbness or prickling in my hands or feet	27.5	72.5
Diarrhea	25.0	75.0
Hair loss or changes in hair	24.2	75.8
Headache	23.3	76.7
Fever, feeling cold or sweating	19.2	80.8
Coughing or out of breath	18.3	81.6
Loss of appetite or changes in taste	17.5	82.5
Dizzy or light-headed	15.8	84.2
Weight loss or wasting away	15.8	84.1
Nausea or vomiting	13.3	86.7

[a] *Bothersome comprise of responses of "2-a little bothersome", "3-very bothersome", and "4-extremely bothersome"*

[b] *Not bothersome comprised of responses of "0-not bothersome" and "1-not bothersome"*

Supplementary Table 2. Regression model of PLWH 's characteristics and Family support (Total n=119)

Factor		Logistic regression				Multiple logistic regression			
		Crude OR	95% C.I.		*p-value*	Adjusted OR	95% C.I.		*p-value*
			Lower	Upper			Lower	Upper	
**Age**		1.0	1.0	1.0	0.785				
**Gender**	*Male*	ref.							
	Female	0.9	0.2	4.7	0.916				
**Residential type**	*Rural*	ref.							
	Urban	0.9	0.4	2.2	0.799				
**Highest education level**	*Junior high school or less*	ref.							
	Senior high school	1.2	0.3	4.6	0.826				
	University or above	1.3	0.4	4.3	0.619				
**Employment status**	*Unemployed (Home keeping/Retired/Student/Declined to answer)*	ref.							
	Employed (Full-time/Self-employed/Part-time)	1.4	0.7	3.2	0.369				
**Marital status**	*Single/Divorced/Separated/Widowed/Declined to answer*	ref.							
	Married/Living with partner	12.1	1.5	96.9	0.019	12.8	1.6	103.8	0.017
**Annual income (P1Y)**	*Less than USD 16,904/Decline to answer*	ref.							
	Above USD 16,904 ~ USD 50,713	2.5	1.0	6.2	0.040	2.7	1.1	6.7	0.034
**Disease duration**	*2-5 years*	ref.							
	5-10 years	0.4	0.1	1.1	0.082				
	Over 10 years	1.3	0.6	3.1	0.546				
**ART duration**		1.1	1.0	1.1	0.083				
**Number of Comorbidity**		0.8	0.6	1.1	0.225				

**1 patient who declined to answer his/her gender was excluded.*

**ref.: reference group.*

P1Y: past one year

Supplementary Table 3. Regression model of PLWH 's characteristics and Peer support (Total n=119)

Factor		Logistic regression				Multiple logistic regression			
		Crude OR	95% C.I.		*p-value*	Adjusted OR	95% C.I.		*p-value*
			Lower	Upper			Lower	Upper	
**Age**		1.0	1.0	1.0	0.638				
**Gender**	*Male*	ref.							
	Female	0.7	0.1	3.8	0.718				
**Residential type**	*Rural*	ref.							
	Urban	0.7	0.3	1.9	0.497				
**Highest education level**	*Junior high school or less*	ref.							
	Senior high school	2.7	0.6	11.5	0.178	1.1	0.3	4.6	0.855
	University or above	3.7	1.0	12.9	0.043	1.1	0.3	3.6	0.887
**Employment status**	*Unemployed (Home keeping/Retired/Student/Declined to answer)*	ref.							
	Employed (Full-time/Self-employed/Part-time)	2.7	1.2	6.1	0.016	1.1	0.5	2.6	0.751
**Marital status**	*Single/Divorced/Separated/Widowed/Declined to answer*	ref.							
	Married/Living with partner	0.7	0.2	2.4	0.599				
**Annual income (P1Y)**	*Less than USD 16,904/Decline to answer*	ref.							
	Above USD 16,904 ~ USD 50,713	3.0	1.2	7.7	0.023	2.4	0.9	6.3	0.065
**Disease duration**	*2-5 years*	ref.							
	5-10 years	0.7	0.2	1.8	0.428				
	Over 10 years	1.0	0.4	2.3	0.919				
**ART duration**		1.0	0.9	1.1	0.956				
**Number of Comorbidity**		0.9	0.6	1.2	0.369				

**1 patient who declined to answer his/her gender was excluded.*

**ref.: reference group.*

P1Y: past one year

Supplementary Table 4. Regression model of PLWH 's characteristics and Social support (Total n=119)

Factor		Logistic regression				Multiple logistic regression			
		Crude OR	95% C.I.		*p-value*	Adjusted OR	95% C.I.		*p-value*
			Lower	Upper			Lower	Upper	
**Age**		1.0	1.0	1.1	0.238				
**Gender**	*Male*	ref.							
	Female	1.8	0.3	9.1	0.502				
**Residential type**	*Rural*	ref.							
	Urban	0.6	0.3	1.7	0.364				
**Highest education level**	*Junior high school or less*	ref.							
	Senior high school	2.2	0.5	9.5	0.271				
	University or above	1.2	0.3	4.2	0.790				
**Employment status**	*Unemployed (Home keeping/Retired/Student/Declined to answer)*	ref.							
	Employed (Full-time/Self-employed/Part-time)	1.0	0.4	2.2	0.980		NA		
**Marital status**	*Single/Divorced/Separated/Widowed/Declined to answer*	ref.							
	Married/Living with partner	1.2	0.4	4.2	0.723				
**Annual income (P1Y)**	*Less than USD 16,904/Decline to answer*	ref.							
	Above USD 16,904 ~ USD 50,713	1.1	0.4	2.5	0.902				
**Disease duration**	*2-5 years*	ref.							
	5-10 years	0.4	0.1	1.3	0.120				
	Over 10 years	1.1	0.5	2.7	0.788				
**ART duration**		1.0	0.9	1.1	0.972				
**Number of Comorbidity**		1.0	0.7	1.4	0.918				

**1 patient who declined to answer his/her gender was excluded.*
**ref.: reference group.*
P1Y: past one year

Supplementary Table 5. Demographics of PLWH and (pre-matched and post-matched) non-PLWH respondents

	Pre-matched			Post-matched*		
	**PLWH**	**Non-PLWH**	***p-value***	**PLWH**	**Non-PLWH**	***p-value***
**n**	**119**	**19964**		**119**	**119**	
Mean Age (SD)	39.68 (9.98)	46.49 (15.62)	<0.001	39.68 (9.98)	40.81 (12.70)	0.448
Gender n, (%)						
Male	113 (95.0)	9680 (48.5)	<0.001	113 (95.0)	112 (94.1)	1
Female	6 (5.0)	10284 (51.5)		6 (5.0)	7 (5.9)	
Highest education level n, (%)						
Junior high school or less	13 (10.9)	1663 (8.3)	0.003	13 (10.9)	9 (7.6)	0.598
Senior high school/vocational school	22 (18.5)	6548 (32.8)		22 (18.5)	20 (16.8)	
University or above	84 (70.6)	11445 (57.3)		84 (70.6)	90 (75.6)	
Decline to answer	0 (0.0)	308 (1.5)		0 (0.0)	0 (0.0)	
Marital status n, (%)						
Married/living with partner	12 (10.1)	11049 (55.3)	<0.001	12 (10.1)	10 (8.4)	0.809
Single/Divorced/Widowed	104 (87.4)	8814 (44.1)		104 (87.4)	107 (89.9)	
Decline to answer	3 (2.5)	101 (0.5)		3 (2.5)	2 (1.7)	
Income level n, (%)						
Low	81 (68.1)	15560 (77.9)	<0.001	81 (68.1)	80 (67.2)	0.817
Middle	29 (24.4)	2392 (12.0)		29 (24.4)	32 (26.9)	
High	0 (0.0)	924 (4.6)		0 (0.0)	0 (0.0)	
Decline to answer	9 (7.6)	1088 (5.4)		9 (7.6)	7 (5.9)	
Residential type n, (%)						
Urban	97 (81.5)	16189 (81.1)	1	97(81.5)	101 (84.9)	0.603
Rural	22 (18.5)	3775 (18.9)		22 (18.5)	18 (15.1)	

**Using propensity score matching (PSM), 1 to 1 matched.*
